# Nogo Receptor Antagonist LOTUS Promotes Neurite Outgrowth through Its Interaction with Teneurin-4

**DOI:** 10.3390/cells13161369

**Published:** 2024-08-17

**Authors:** Yuji Kurihara, Yuki Kawaguchi, Yuki Ohta, Nana Kawasaki, Yuki Fujita, Kohtaro Takei

**Affiliations:** 1 Molecular Medical Bioscience Laboratory, Department of Medical Life Science, Yokohama City University Graduate School of Medical Life Science, Yokohama 230-0045, Japan; 2 Department of Anatomy & Developmental Biology, Faculty of Medicine, Shimane University, Izumo 693-8501, Japan; 3 Department of Regenerative Medicine, Yokohama City University Graduate School of Medicine, Yokohama 230-0045, Japan; 4 Laboratory of Biopharmaceutical and Regenerative Sciences, Department of Medical Life Science, Yokohama City University Graduate School of Medical Life Science, Yokohama 230-0045, Japan

**Keywords:** lateral olfactory tract usher substance, neurite outgrowth, Teneurin-4

## Abstract

**Simple Summary:**

Neuronal growth is an essential process for establishing functional neural networks in developing brain and restoring dysfunctional networks caused by injuries in the adult brain. In the adult central nervous system, injured neurons fail to re-extend their axons due to several axon growth inhibitors mainly expressed in glial cells. We previously reported that LOTUS protein helps injured adult neurons to regenerate by blocking the inhibitors. In this study, we found that LOTUS itself promotes axon growth through an independent mechanism from the blockade of the inhibitors. LOTUS directly binds to Teneurin-4, a cell adhesion molecule expressed on the surface of neurons and the binding promotes axon growth. Thus, LOTUS was found to have two functions in axon growth. This finding may provide a new insight into neural network formation in developing brain and neural regrowth mechanism following the adult injured brain. These functions of LOTUS protein are useful for developing new therapeutic approaches to promote nerve regeneration after injury in the adult central nervous system.

**Abstract:**

Neurite outgrowth is a crucial process for organizing neuronal circuits in neuronal development and regeneration after injury. Regenerative failure in the adult mammalian central nervous system (CNS) is attributed to axonal growth inhibitors such as the Nogo protein that commonly binds to Nogo receptor-1 (NgR1). We previously reported that lateral olfactory tract usher substance (LOTUS) functions as an endogenous antagonist for NgR1 in forming neuronal circuits in the developing brain and improving axonal regeneration in the adult injured CNS. However, another molecular and cellular function of LOTUS remains unknown. In this study, we found that cultured retinal explant neurons extend their neurites on the LOTUS-coating substrate. This action was also observed in cultured retinal explant neurons derived from *Ngr1*-deficient mouse embryos, indicating that the promoting action of LOTUS on neurite outgrowth may be mediated by unidentified LOTUS-binding protein(s). We therefore screened the binding partner(s) of LOTUS by using a liquid chromatography-tandem mass spectrometry (LC-MS/MS). LC-MS/MS analysis and pull-down assay showed that LOTUS interacts with Teneurin-4 (Ten-4), a cell adhesion molecule. RNAi knockdown of *Ten-4* inhibited neurite outgrowth on the LOTUS substrate in retinoic acid (RA)-treated Neuro2A cells. Furthermore, a soluble form of Ten-4 attenuates the promoting action on neurite outgrowth in cultured retinal explant neurons on the LOTUS substrate. These results suggest that LOTUS promotes neurite outgrowth by interacting with Ten-4. Our findings may provide a new molecular mechanism of LOTUS to contribute to neuronal circuit formation in development and to enhance axonal regeneration after CNS injury.

## 1. Introduction

During development, post-mitotic neurons establish functional wiring of neuronal circuits by migrating to their precise locations, extending their neurites to precise target cells and forming synapses with the appropriate partners. Neurite outgrowth, one of these processes, is controlled by extracellular matrix molecules, neurotrophic factors, and cell adhesion molecules [1,2,3,4,5,6]. Teneurin-4 (Ten-4), which is a Teneurin family member and one of four types categorized as cell-adhesion molecules [7], is a transmembrane glycoprotein that induces cell adhesion by interacting with each of the four Teneurin family members [8]. Ten-4 is expressed in various tissues in developmental stages [9,10,11,12] including a high expression in developing central nervous systems (CNSs) [12]. Ten-4 is also expressed in postnatal and adult CNSs [10,11,12,13] and is localized to neurons and oligodendrocytes in postnatal and adult spinal cords [13]. Embryonic, postnatal, and adult CNSs express the other Teneurin family members: Ten-1, Ten-2, and Ten-3 [9,10,11,12]. Ten-4 transduces the signal to focal adhesion kinase (FAK), which is an intracellular tyrosine kinase that regulates cell adhesion and migration by mediating the signals transduced by integrins and growth factor receptors [14], leading to the promotion of neurite outgrowth [15]. These previous reports suggest that Ten-4 could mediate neurite outgrowth through homophilic or heterophilic interaction with each of the four Teneurin family members expressed in other neural cells.

We previously identified lateral olfactory tract usher substance (LOTUS), which is a membrane protein and secretory protein that is mainly expressed in the CNS [16,17,18,19,20], as a novel key molecule that contributes to axonal bundling formation of the lateral olfactory tract through its antagonism of Nogo receptor-1 (NgR1) [18]. NgR1 is a common receptor for the five axonal growth inhibitors such as Nogo-A, myelin-associated glycoprotein (MAG), oligodendrocyte myelin glycoprotein (OMgp), B lymphocyte stimulator, and chondroitin sulfate proteoglycans, and it mediates axonal growth inhibition caused by these five inhibitors [21,22,23,24,25]. LOTUS performs a suppressive activity on the axonal growth inhibition that each of these five inhibitors cause through their binding to NgR1 [18,26,27]. Recently, we have shown that LOTUS exerts an antagonism for paired immunoglobulin-like receptor (PIR)-B [28], which is another receptor for Nogo-A, MAG, and OMgp and mediates axonal growth inhibition induced by these three PIR-B-ligands [29]. Thus, LOTUS is an antagonist for both receptors of NgR1 and PIR-B. However, it remains unknown whether LOTUS has another molecular and cellular function in developmental and regenerative phenomena. Here, we report that LOTUS has a neurite outgrowth activity, and this activity is independent of the antagonism for NgR1. Furthermore, we show that Ten-4 is identified as a new LOTUS-binding protein, and *Ten-4*-knockdown and a soluble form of Ten-4 attenuate LOTUS-induced neurite outgrowth activity. Our data suggest that LOTUS promotes neurite outgrowth by interacting with Ten-4.

## 2. Materials and Methods

### 2.1. Animals

Wild-type C57BL/6J mice were obtained commercially from Japan SLC (Hamamatsu, Japan). The *Ngr1*-knockout mice [30] were gifted by Dr. Stephen M. Strittmatter at Yale University. These mice were bred in a specific-pathogen-free (SPF) facility under light/dark cycles (12 h/12 h) and provided autoclaved water and food pellets ad libitum. The genotypes of the mutant mice and mouse embryos were determined by PCR using the primers to amplify the *neomycin* gene (5′-cta ttc ggc tat gac tgg gca caa cag ac-3′/5′-gaa ctc gtc aag aag gcg ata gaa ggc gat-3′) or the mouse *Ngr1* exon 2 (5′-cag tac ctg cga ctc aat gac aac ccc-3′/5′-ctt ccg gga aca acc tgg cct cc-3′).

Throughout the experimental procedures, all efforts were made to minimize the number of animals used. The adult mice used were treated with isoflurane (099-06571, Wako Pure Chemical Industries, Osaka, Japan) and humanely euthanized. The liver, the embryonic brain, or the embryonic retinae were extracted and used for each experiment. The experimental protocols were approved by the Institutional Animal Care and Use Ethics Committee of Yokohama City University and by the Animal Care and Use Committee of Shimane University.

### 2.2. Antibodies and Reagents

Anti-rat LOTUS mouse monoclonal antibodies (custom-made, ITM, Matsumoto, Japan), anti-human Ten-4 sheep polyclonal antibodies (AF6320, R&D Systems, Minneapolis, MN, USA), anti-α-tubulin mouse monoclonal antibodies (sc-32293, Santa Cruz Biotechnology, Dallas, TX, USA), anti-βIII-tubulin rabbit polyclonal antibodies (802001, BioLegend, San Diego, CA, USA), anti-β-tubulin mouse monoclonal antibodies (014-25041, Wako Pure Chemical Industries), Alexa Fluor 488-labeled anti-mouse IgG goat antibodies (A11001, Invitrogen, Waltham, MA, USA), Cy3-labeled anti-rabbit IgG goat antibodies (111-165-003, Jackson ImmunoResearch, West Grove, PA, USA), Cy3-labeled anti-mouse IgG donkey antibodies (715-165-151, Jackson ImmunoResearch), horseradish peroxidase (HRP)-labeled anti-mouse IgG goat antibodies (115-035-003, Jackson ImmunoResearch), and HRP-labeled anti-sheep IgG donkey antibodies (713-035-147, Jackson ImmunoResearch) were purchased.

### 2.3. Construction of Expression Plasmids

The plasmids expressing the signal peptide sequence-deleted mouse LOTUS having streptavidin-binding protein (SBP)-and FLAG-tag at the N terminus (SBP-FLAG-LOTUS) and SBP-fused mutated human Fc (SBP-Fc) were described as previously reported [28,31]. The sequence coding full-length mouse Ten-4 (NM_011858) was amplified using cDNA derived from an adult mouse brain and inserted into a mammalian expression vector. The expression plasmids of mouse Ten-4 tagging HA (Ten-4-HA) and SBP-Fc-fused extracellular domains of Ten-4 (SBP-Fc-Ten-4) were generated. DNA sequencing confirmed all of the nucleotide sequences inserted into each expression plasmid.

### 2.4. Protein Purification

HEK293T cells were plated on 145-mm dishes for cell culture (9 × 10^6^ cells/dish) (639160, Greiner Bio-One, Kremsmünster, Austria) and grown for 2 d in Dulbecco’s modified eagle’s medium (DMEM) (08458-16, Nacalai Tesque, Kyoto, Japan) supplemented with a 0.5% penicillin-streptomycin mixed solution (26253-84, Nacalai Tesque) and 10% fetal bovine serum (FBS) (S1400-500, Biowest, Nuaillé, France). The grown cells were transfected with the expression plasmid of SBP-Fc or SBP-Fc-Ten-4 using a transfection reagent (Polyethylenimine Max, 24765-100, Polysciences, Warrington, PA, USA) and grown further for 4 d. HEK293 cells stably overexpressing His-tagged ectodomains of human placental alkaline phosphatase (AP) (His-AP), His-AP-fused mouse LOTUS deleting its signal peptide sequence (His-AP-LOTUS), or His-AP-fused mouse LOTUS deleting its signal peptide sequence and its four phenylalanyl-glycyl and glycyl-alanyl-prolyl domains (His-AP-UA/EC) were plated on 145-mm dishes for cell culture (639160, Greiner Bio-One) and grown for 1 w in DMEM (08458-16, Nacalai Tesque) supplemented with a 0.5% penicillin-streptomycin mixed solution (26253-84, Nacalai Tesque) and 10% FBS (S1820-500, Biowest) (S1400-500, Biowest) and further for 1 w in the fresh DMEM (08458-16, Nacalai Tesque) supplemented with a 0.5% penicillin-streptomycin mixed solution (26253-84, Nacalai Tesque) and 10% FBS (S1820-500, Biowest) (S1400-500, Biowest). The conditioned medium was ultracentrifuged, and the centrifuged supernatant including SBP-tagged protein was applied to streptavidin resins (High-Capacity Streptavidin Agarose Resin, 20361, Thermo Fisher Scientific, Waltham, MA, USA). The centrifuged supernatant including His-tagged protein was dialyzed with 20 mM Tris-Cl pH 7.6 and 500 mM NaCl and then applied to cobalt resins (TALON Metal Affinity Resin, 635501, Clontech Laboratories, CA, USA). SBP-tagged proteins were eluted from the resins with phosphate-buffered saline (PBS) including 2 mM biotin. His-tagged proteins were eluted from the resins with the above dialysis solution including 50 mM imidazole. The eluted proteins were stored at −80 °C until use.

AP-fused proteins were incubated with *p*-nitrophenyl phosphate (*p*NPP) (N2770-50SET, Sigma-Aldrich, St. Louis, MO, USA), and then the absorbance at 405 nm was measured using xMark Microplate Absorbance Spectrophotometer (Bio-Rad, Hercules, CA, USA) or DTX 880 Multimode Detector (Beckman Coulter, Brea, CA, USA). Non-AP-fused proteins were incubated with a 4× Laemmli buffer (250 mM Tris-Cl pH 6.8, 8% SDS, 40% glycerol, and 0.06% bromophenol blue) including 10% 2-mercaptoethanol and boiled for 7 min. The proteins were separated by SDS-PAGE and stained with Coomassie brilliant blue R-250 (031-17922, Wako Pure Chemical Industries). The staining bands were detected with a WSE-5300 Printgraph CMOS I (ATTO corporation, Tokyo, Japan) and quantified using ImageJ software (version 1.41, National Institutes of Health, Bethesda, MD, USA).

### 2.5. Ligand Binding Assay

Poly-l-lysine (100 μg/mL, 163-19091, Wako Pure Chemical Industries) and mouse laminin (10 μg/mL, 23017-015, Invitrogen) were sequentially coated to a multi-well plate for cell culture (176740, Thermo Fisher Scientific). Neuro2A cells were plated on the coated plates (3 × 10^4^ cells/well) and grown for 16 h in minimal essential medium (MEM) (21443-15, Nacalai Tesque) supplemented with 10% FBS (S1820-500, Biowest) and further for 48 h in MEM (21443-15, Nacalai Tesque) supplemented with 20 μM retinoic acid (RA) (186-01114, Wako Pure Chemical Industries) and 2% FBS (S1820-500, Biowest). The grown cells were treated with His-AP or His-AP-LOTUS (25 nM) in the fresh MEM (21443-15, Nacalai Tesque) supplemented with 2% FBS (S1820-500, Biowest) and fixed with warmed 4% paraformaldehyde in PBS including 2 mM MgCl_2_ for 10 min at 37 °C and then for 10 min at room temperature. The fixed cells were incubated with 2 mM MgCl_2_ in PBS for 1 h at 67 °C, and then with nitro blue tetrazolium (NBT) (11383213001, Roche, Basel, Switzerland) and 5-bromo-4-chloro-3-indolyl phosphate (BCIP) (11383221001, Roche) to visualize the binding of the protein to the cells. The digital images were acquired using a microscope (Olympus IX71, Olympus, Tokyo, Japan) with a 20× objective lens and a CCD camera (Olympus DP72, Olympus).

### 2.6. Purification of Proteins Interacting with LOTUS

Neuro2A cells were plated on 145-mm dishes for cell culture (3 × 10^6^ cells/dish) (639160, Greiner Bio-One), grown for 16 h in MEM (21443-15, Nacalai Tesque) supplemented with a 0.5% penicillin-streptomycin mixed solution (26253-84, Nacalai Tesque) and 10% FBS (S1820-500, Biowest) and transfected with the SBP-FLAG-LOTUS expression plasmid using a transfection reagent (Lipofectamine 3000 Transfection Reagent, L3000015, Invitrogen). The transfected cells were grown for 48 h and incubated with a lysis solution composed of 137 mM NaCl, 20 mM Tris-Cl pH 7.5, 1 mM EDTA-Na pH 8.0, 0.05 mM (*p*-Amidinophenyl)methanesulfonyl fluoride, 1 mM Na_3_VO_4_ pH 10.0, 10 mM NaF, a protease inhibitor cocktail (cOmplete, Mini, 11836170001, Roche), and 1% Nonidet P-40. The lysates were centrifuged at 16,000 *g* for 10 min, and the centrifuged supernatant was applied to streptavidin resins (Streptavidin Sepharose High Performance, 17511301, GE Healthcare Life Sciences, Buckinghamshire, UK). The resins were incubated with a 4× Laemmli buffer (250 mM Tris-Cl pH 6.8, 8% SDS, 40% glycerol, and 0.06% bromophenol blue) including 10% 2-mercaptoethanol, boiled for 7 min and stored at −80 °C until use.

### 2.7. In-Gel Digestion

The denatured and reduced proteins were separated by SDS-PAGE and stained with SYPRO Ruby Protein Gel Stain (S12001, Invitrogen). The reactive bands were excised from the gel and cut into several pieces. The gel pieces were decolorized with a 50 mM ammonium bicarbonate solution including 50% methanol for 20 min at room temperature, dehydrated with acetonitrile, and dried by vacuum centrifugation for 16 h at room temperature. The samples in the dried pieces were reduced with 10 mM dithiothreitol in a 25 mM ammonium bicarbonate solution for 30 min at 65 °C and alkylated with 55 mM iodoacetamide in a 25 mM ammonium bicarbonate solution for 45 min at room temperature, and then the pieces were dehydrated with acetonitrile and dried by vacuum centrifugation for 2 h at room temperature. The redried pieces were incubated sequentially with 20 ng/μL Trypsin (Trypsin/Lys-C Mix, Mass Spec Grade, V5072, Promega, Madison, WI, USA) in a 25 mM ammonium bicarbonate solution for 30 min at 37 °C and with a Trypsin-free 25 mM ammonium bicarbonate solution for 16 h at 37 °C. The supernatant of Trypsin-treated gel pieces was collected and the gel pieces were incubated sequentially with 10% acetonitrile for 10 min at room temperature, 30% acetonitrile for 10 min at room temperature, 50% acetonitrile for 10 min at room temperature, and ultrapure water for 5 min at room temperature and subsequently 50% acetonitrile for 15 min at room temperature. All of the supernatants of the gel pieces were pooled, dried by vacuum centrifugation for 16 h at room temperature, and stored at −80 °C until use.

### 2.8. Liquid Chromatography-Tandem Mass Spectrometry

The dried samples were dissolved with ultrapure water, and then the solution was applied to a C18 reversed-phase column embedded into a micropipette tip. The peptides were eluted with a 45% acetonitrile solution including 0.1% trifluoroacetic acid, dried by vacuum centrifugation at room temperature, and then dissolved with a 0.1% formic acid solution. The dissolved peptides were analyzed by liquid chromatography-tandem mass spectrometry (LC-MS/MS) using an Orbitrap mass spectrometer (Q Exactive Hybrid Quadrupole-Orbitrap Mass Spectrometer, IQLAAEGAAPFALGMAZR, Thermo Fisher Scientific) interfaced on-line with a nano-flow liquid chromatography (EASY-nLC 1000 Liquid Chromatograph, LC-120, Thermo Fisher Scientific) equipped with a trap column (0.075 mm i.d, 20 mm length, 3 μm particle size, Acclaim PepMap 100 C18 HPLC Columns, 164946, Thermo Fisher Scientific) and an analytical column (0.075 mm i.d, 125 mm length, 3 μm particle size, 3 μm C18 Nano HPLC capillary column 75-3-12, NTCC-360/75-3-125, Nikkyo Technos, Tokyo, Japan) at a flow rate of 300 nL/min with three-step linear gradients from 0–40% mobile phase B for 60 min, 40–100% mobile phase B for 1 min, and 100% mobile phase B for 5 min. The Mobile phase B consisted of 100% acetonitrile including 0.1% formic acid, whereas the mobile phase A consisted of water including 0.1% formic acid. Data-dependent MS/MS acquisitions were performed for the most intense precursor ions. Peptides and proteins were identified by searching the MS/MS spectra against a mouse protein database (UniProt) using proteomic data analysis software (Thermo Proteome Discoverer version 1.4, Thermo Fisher Scientific).

### 2.9. Pull-Down Assay

COS7 cells were plated on a multi-well plate for cell culture (5 × 10^5^ cells/well) (657160, Greiner Bio-One), grown for 4 h in DMEM (08458-16, Nacalai Tesque) supplemented with a 0.5% penicillin-streptomycin mixed solution (26253-84, Nacalai Tesque) and 10% FBS (S1820-500, Biowest) and transfected with the plasmid overexpressing SBP-FLAG-LOTUS and/or Ten-4-HA using a transfection reagent (FuGENE 6 Transfection Reagent, E2692, Roche). The transfected cells were grown for 44 h and incubated with a lysis solution composed of 137 mM NaCl, 20 mM Tris-Cl pH 7.5, 1 mM EDTA-Na pH 8.0, 0.05 mM (*p*-Amidinophenyl)methanesulfonyl fluoride, 1 mM Na_3_VO_4_ pH 10.0, 10 mM NaF, a protease inhibitor cocktail (cOmplete, Mini, 11836170001, Roche), and 1% Nonidet P-40. The lysates were centrifuged at 16,000 *g* for 10 min, and the centrifuged supernatant was applied to streptavidin resins (Streptavidin Sepharose High Performance, 17511301, GE Healthcare Life Sciences). The resins were incubated with a 4× Laemmli buffer (250 mM Tris-Cl pH 6.8, 8% SDS, 40% glycerol, and 0.06% bromophenol blue) including 10% 2-mercaptoethanol, boiled for 7 min and stored at −80 °C until use.

### 2.10. Western Blotting

The denatured and reduced proteins were separated by SDS-PAGE and transferred onto a polyvinylidene fluoride membrane (Immobilon-P Membrane, IPVH00010, Millipore, Burlington, MA, USA). The membrane was cut and blocked with 1% or 5% skim milk in Tris-buffered saline (TBS) including 0.1% Tween 20 for 1 h at room temperature, incubated sequentially with anti-Ten-4 antibodies (0.04 μg/mL, AF6320, R&D Systems) in 1% skim milk in TBS including 0.1% Tween 20 overnight at 4 °C or with anti-LOTUS antibodies (0.42 μg/mL, custom-made, ITM) in 5% bovine serum albumin (BSA) in TBS including 0.1% Tween 20 overnight at 4 °C and with HRP-labeled anti-sheep IgG antibodies (0.16 μg/mL, 713-035-147, Jackson ImmunoResearch) in 1% skim milk in TBS including 0.1% Tween 20 for 1 h at room temperature or with HRP-labeled anti-mouse IgG antibodies (0.16 μg/mL, 115-035-003, Jackson ImmunoResearch) in 5% skim milk in TBS including 0.1% Tween 20 for 1 h at room temperature. The treated membrane was incubated with a chemiluminescent HRP substrate (Immobilon Western Chemiluminescent HRP Substrate, WBKLS0100, Millipore) (ECL Western Blotting Detection Reagents, RPN2109, GE Healthcare Life Sciences). The digital images were acquired with LAS-4000 multi color apparatus (Fujifilm, Tokyo, Japan) and ImageQuant TL software v10 (GE Healthcare Life Sciences).

### 2.11. RNA Isolation and RT-PCR

Neuro2A cells were plated on 35-mm dishes for cell culture (4 × 10^5^ cells/dish) (VTC-D35N, AS ONE Corporation, Osaka, Japan), grown for 14 h in MEM (21443-15, Nacalai Tesque) supplemented with 10% FBS (S1400-500, Biowest), transfected with dicer-substrate short interfering RNA (DsiRNA) designed not to target any genes (Negative Control DsiRNA, 51-01-14-03, Integrated DNA Technologies, Coralville, IA, USA) or designed to target mouse *Ten-4* mRNA (mm.Ri.Tenm4.13.1, #1, Integrated DNA Technologies) (mm.Ri.Tenm4.13.2, #2, Integrated DNA Technologies) (mm.Ri.Tenm4.13.3, #3, Integrated DNA Technologies) using a transfection reagent (Lipofectamine RNAiMAX Transfection Reagent, 13778030, Thermo Fisher Scientific) and grown further for 4 h. The transfected cells were grown for 48 h in the fresh MEM (21443-15, Nacalai Tesque) supplemented with 20 μM RA (186-01114, Wako Pure Chemical Industries), a 0.5% penicillin-streptomycin mixed solution (26253-84, Nacalai Tesque) and 2% FBS (S1400-500, Biowest). Total RNAs were isolated from the cultured Neuro2A cells, the liver in wild-type adult mice or the brain or retinae in wild-type mouse embryos at embryonic day 13 (E13) using TRIZOL Reagent (15596-018, Thermo Fisher Scientific) and reverse-transcribed into cDNA with PrimeScript FAST RT reagent Kit with gDNA Eraser (RR092S, Takara Bio, Kusatsu, Japan). The synthesized cDNAs were amplified with a DNA polymerase (TB Green Premix Ex Taq II FAST qPCR, RR830A, Takara Bio) using the following primers to detect the mouse mRNA sequence of *Beta-actin* (5′-cat ccg taa aga cct cta tgc caa c-3′/5′-atg gag cca ccg atc cac a-3′) or *Ten-4* (#1, 5′-gta tct gga ttc agg aat ctg g-3′/5′-atg cag tca ccg tta cca tag-3′) (#2, 5′-aag cca gac aca gat gcc at-3′/5′-ggt caa agc gtt cta agg taa cg-3′), and measured using a quantitative PCR system (CronoSTAR™ 96 Real-Time PCR System, 640231, Takara Bio), and/or amplified with a DNA polymerase (GoTaq Green Master Mix 2X, M7123, Promega) using the above primer sequences. The amplified PCR products were electrophoresed on 1.5% agarose gel and stained with ethidium bromide. The digital images were acquired with WSE-5300 Printgraph CMOS I (ATTO corporation).

### 2.12. Neurite Outgrowth Assay for Neuro2A Cells

Poly-l-lysine (100 μg/mL, 163-19091, Wako Pure Chemical Industries) and His-AP or His-AP-LOTUS (25 nM) were sequentially coated to a multi-well plate for cell culture (176740, Thermo Fisher Scientific). Neuro2A cells were plated on the coated plate (3 × 10^4^ cells/well) and grown overnight in MEM (21443-15, Nacalai Tesque) supplemented with 10% FBS (S1820-500, Biowest) and further for 48 h in MEM (21443-15, Nacalai Tesque) supplemented with 20 μM RA (186-01114, Wako Pure Chemical Industries) and 2% FBS (S1820-500, Biowest). The grown cells were fixed with warmed 4% paraformaldehyde in the conditioned medium for 10 min at 37 °C and then for 10 min at room temperature. The fixed cells were treated with PBS including 1% BSA and 0.1% Triton X-100 for 1 h at room temperature, and immunostained sequentially with anti-α-tubulin antibodies (0.2 μg/mL, sc-32293, Santa Cruz Biotechnology) in the blocking and permeabilizing buffer for 1 h at room temperature and with Alexa Fluor 488-labeled anti-mouse IgG antibodies (2 μg/mL, A11001, Invitrogen) in the blocking and permeabilizing buffer for 1 h at room temperature.

Neuro2A cells were collected with Trypsin treatment (32777-44, Nacalai Tesque), transfected with DsiRNA designed not to target any genes (Negative Control DsiRNA, 51-01-14-03, Integrated DNA Technologies) or designed to target mouse *Protocadherin-16* mRNA (mm.Ri.Dchs1.13.1, Integrated DNA Technologies) (mm.Ri.Dchs1.13.2, Integrated DNA Technologies) (mm.Ri.Dchs1.13.3, Integrated DNA Technologies) or mouse *Ten-3* mRNA (mm.Ri.Tenm3.13.1, Integrated DNA Technologies) (mm.Ri.Tenm3.13.2, Integrated DNA Technologies) (mm.Ri.Tenm3.13.3, Integrated DNA Technologies) using an electroporator (Super Electroporator NEPA21 Type II, Nepa Gene corporation, Ichikawa, Japan), plated on the coated plate (1 × 10^4^ cells/well) and grown for 24 h in MEM (21443-15, Nacalai Tesque) supplemented with a 0.5% penicillin-streptomycin mixed solution (26253-84, Nacalai Tesque) and 10% FBS (S1820-500, Biowest) and further for 48 h in the fresh MEM (21443-15, Nacalai Tesque) supplemented with 20 μM RA (186-01114, Wako Pure Chemical Industries), a 0.5% penicillin-streptomycin mixed solution (26253-84, Nacalai Tesque), and 2% FBS (S1820-500, Biowest). The grown cells were fixed with warmed 4% paraformaldehyde in the conditioned medium for 10 min at 37 °C and then for 10 min at room temperature. The fixed cells were treated with PBS including 0.1% Triton X-100 for 30 min at room temperature, and immunostained sequentially with anti-βIII-tubulin antibodies (0.2 μg/mL, 802001, BioLegend) in TBS including 0.1% Tween 20 for 1 h at room temperature and with Cy3-labeled anti-rabbit IgG antibodies (0.75 μg/mL, 111-165-003, Jackson ImmunoResearch) in TBS including 0.1% Tween 20 for 1 h at room temperature.

Neuro2A cells were plated on the coated plate (1 × 10^4^ cells/well), grown for 14 h in MEM (21443-15, Nacalai Tesque) supplemented with 10% FBS (S1400-500, Biowest), transfected with DsiRNA designed not to target any genes (Negative Control DsiRNA, 51-01-14-03, Integrated DNA Technologies) or designed to target mouse *Ten-4* mRNA (mm.Ri.Tenm4.13.1, #1, Integrated DNA Technologies) (mm.Ri.Tenm4.13.2, #2, Integrated DNA Technologies) (mm.Ri.Tenm4.13.3, #3, Integrated DNA Technologies) using a transfection reagent (Lipofectamine RNAiMAX Transfection Reagent, 13778030, Thermo Fisher Scientific) and grown further for 4 h. The transfected cells were grown further for 48 h in the fresh MEM (21443-15, Nacalai Tesque) supplemented with 20 μM RA (186-01114, Wako Pure Chemical Industries), a 0.5% penicillin-streptomycin mixed solution (26253-84, Nacalai Tesque), and 2% FBS (S1400-500, Biowest). The grown cells were fixed with warmed 4% paraformaldehyde in the conditioned medium for 10 min at 37 °C and then for 10 min at room temperature. The fixed cells were treated with PBS including 0.1% Triton X-100 for 30 min at room temperature, and immunostained sequentially with anti-β-tubulin antibodies (0.25 μg/mL, 014-25041, Wako Pure Chemical Industries) in TBS including 0.1% Tween 20 for 1 h at room temperature and with Cy3-labeled anti-mouse IgG antibodies (0.75 μg/mL, 715-165-151, Jackson ImmunoResearch) in TBS including 0.1% Tween 20 for 1 h at room temperature.

The digital images were acquired using a microscope (Olympus IX71, Olympus) with a 20× objective lens and a CCD camera (Olympus DP72, Olympus), using a microscope (Eclipse Ti-S/L100, Nikon) with a 4× or 10× objective lens and a CCD camera (DS-Fi2, Nikon), or using a microscope (Olympus IX83, Olympus) with a 4× objective lens and a CCD camera (Olympus DP80, Olympus). The distance from an initial neurite segment to the neurite tip in each cell was measured as the neurite length in the Neuro2A cell using ImageJ software (version 1.41, National Institutes of Health), and the maximum neurite length was shown as the single or longest neurite in each cell.

### 2.13. Neurite Outgrowth Assay for Retinal Ganglion Cells

Poly-l-lysine (100 μg/mL, 163-19091, Wako Pure Chemical Industries) and His-AP, His-AP-LOTUS, or His-AP-UA/EC (25 nM) were sequentially coated to a multi-well plate for cell culture (176740, Thermo Fisher Scientific). Retinae were extracted from wild-type or *Ngr1*-deficient mouse E13 embryos, segmented, plated on the coated plate, and grown for 48 h in DMEM/Ham’s F-12 medium (D8062, Sigma-Aldrich) supplemented with a 0.5% penicillin-streptomycin mixed solution (26253-84, Nacalai Tesque) and 1% N-2 supplement (17502-048, Invitrogen). The grown retinal explants were fixed with warmed 4% paraformaldehyde and 2 mM MgCl_2_ in the conditioned medium for 10 min at 37 °C and then for 10 min at room temperature. The fixed explants were incubated with NBT (11383213001, Roche) and BCIP (11383221001, Roche) and visualized with their enzymatic reaction product by an endogenous AP. The digital images were acquired using a microscope (Olympus IX70, Olympus) with a 4× objective lens and a CCD camera (SPOT-2e, SPOT Imaging, Sterling Heights, MI, USA) or using a microscope (Axiovert 135 M, Zeiss, Oberkochen, Germany) with 4× objective lens and a CCD camera (CoolSnap HQ, Roper Scientific, Tucson, AZ, USA).

Poly-l-lysine (100 μg/mL, 163-19091, Wako Pure Chemical Industries) and His-AP or His-AP-LOTUS (25 nM) pre-incubated with SBP-Fc or SBP-Fc-Ten-4 (1500 nM) were sequentially coated to a multi-well plate for cell culture (176740, Thermo Fisher Scientific). Retinae were extracted from wild-type mouse E13 embryos, segmented, plated on the coated plate and grown for 48 h in DMEM/Ham’s F-12 medium (D8062, Sigma-Aldrich) supplemented with 1% N-2 supplement (17502-048, Invitrogen). The grown retinal explants were fixed with warmed 4% paraformaldehyde in the conditioned medium for 10 min at 37 °C and then for 10 min at room temperature. The fixed explants were treated with PBS including 0.1% Triton X-100 for 30 min at room temperature, and immunostained sequentially with anti-β-tubulin antibodies (0.25 μg/mL, 014-25041, Wako Pure Chemical Industries) in TBS including 0.1% Tween 20 for 1 h at room temperature and with Cy3-labeled anti-mouse IgG antibodies (0.75 μg/mL, 715-165-151, Jackson ImmunoResearch) in TBS including 0.1% Tween 20 for 1 h at room temperature. The digital images were acquired using a microscope (Olympus IX83, Olympus) with a 4× objective lens and a CCD camera (Olympus DP80, Olympus).

The liner distance from the center of an explant to the neurite tip in the outer neurites, minus the radius of the explant was measured as the neurite length of the retinal explant using microscope imaging software (MetaMorph, Molecular Devices, San Jose, CA, USA) or ImageJ software (version 1.41, National Institutes of Health).

### 2.14. Statistical Analysis

The data were presented as mean ± standard error of the mean (SEM) and analyzed by one-way factorial analysis of variance (ANOVA) test followed by post-hoc Tukey–Kramer multiple comparisons test (Tukey–Kramer test), by Kruskal–Wallis test followed by post-hoc Steel multiple comparisons test (Steel test), or by Kruskal–Wallis test followed by post-hoc Steel–Dwass multiple comparisons test (Steel–Dwass test) using a statistical software (BellCurve for Excel version 3.21, Social Survey Research Information, Tokyo, Japan). Statistical significance was defined as *p* < 0.05.

## 3. Results

### 3.1. LOTUS Elongates Neurites in Retinal Explant Neurons

**Figure 1:**
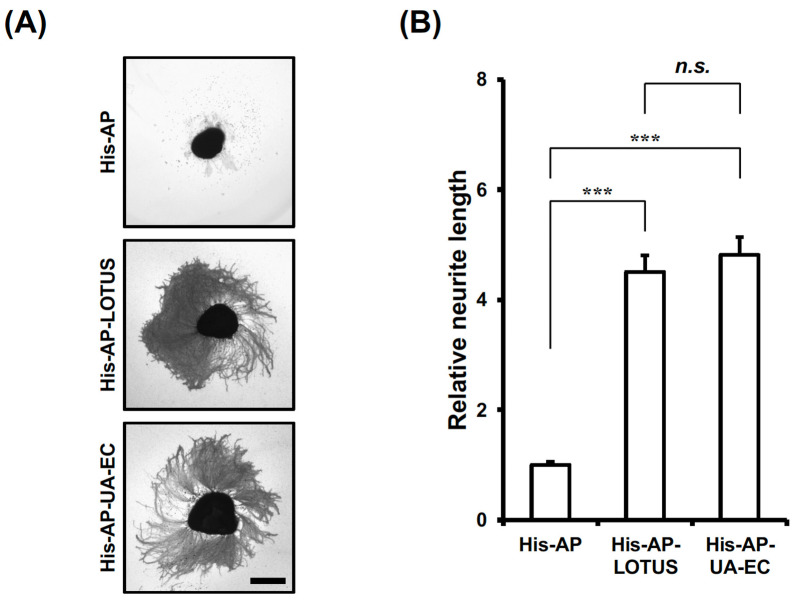
Neurite outgrowth promoted by LOTUS. (**A**) AP staining of endogenous AP in mouse E13 embryonic retinal explant neurons cultured using His-AP, His-AP-LOTUS, or His-AP-UA/EC (25 nM) as a culture substrate. Scale bar, 300 μm. (**B**) Quantification of neurite length in (**A**). The neurite length of the retinal explant was shown as the liner distance from the center of an explant to the neurite tip in the outer neurites, minus the radius of the explant, normalized to the neurite length of the explant cultured on a His-AP substrate and presented as mean ± SEM from five independent cultures (*n* = 15 explants, Kruskal–Wallis test followed by post-hoc Steel–Dwass test; *** *p* < 0.001: n.s. not significant).

We attempted to find another function of LOTUS antagonizing NgR1 and PIR-B [18,26,28]. To examine whether LOTUS itself influences elongation of neurites, we purified LOTUS and the UA/EC domain of LOTUS with a His-AP tag, cultured retinal explants isolated from wild-type mouse E13 embryos, when glial cells are not yet generated in the retina [32], using His-AP-LOTUS or His-AP-UA/EC as a culture substrate, and observed the cultured neurons. The UA/EC domain is the binding and antagonistic domain of LOTUS toward NgR1 [33]. Retinal explant neurons remarkably elongated their neurites on His-AP-LOTUS or His-AP-UA/EC substrate, while no elongating neurites were observed on a His-AP substrate (Figure 1A,B). Thus, LOTUS promoted neurite outgrowth in retinal explant neurons.

### 3.2. LOTUS Also Shows the Similar Neurites-Elongating Activity in Ngr1-Deficient Retinal Explant Neurons

**Figure 2:**
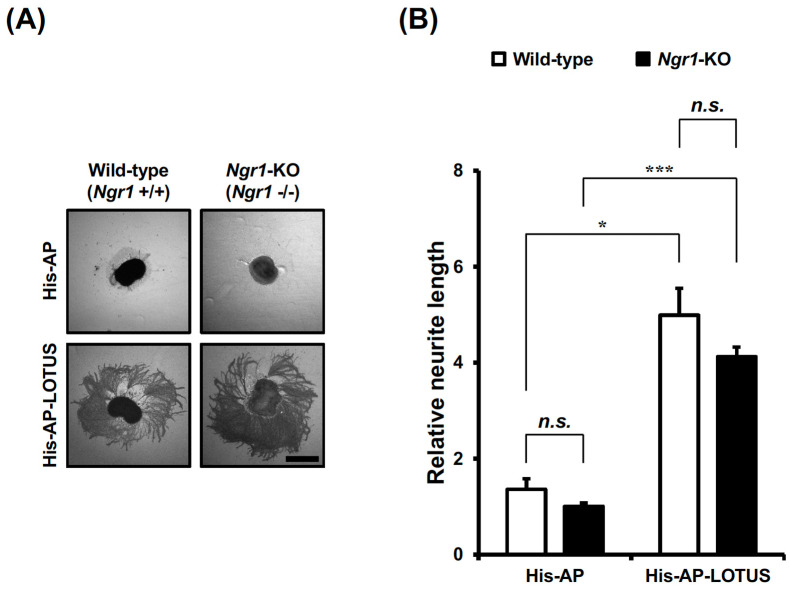
Promoting effect of LOTUS on neurite outgrowth in *Ngr1*-deficient retinal explant neurons. (**A**) AP staining of endogenous AP of *Ngr1*-deficient mouse E13 embryonic retinal explant neurons cultured using His-AP or His-AP-LOTUS (25 nM) as a culture substrate of. Scale bar, 300 μm. (**B**) Quantification of neurite length in (**A**). The neurite length of the retinal explant was shown as the liner distance from the center of an explant to the neurite tip in the outer neurites, minus the radius of the explant, normalized to the neurite length of the explant cultured on a His-AP substrate and presented as mean ± SEM from three independent cultures (*n* = 4–10 explants, Kruskal–Wallis test followed by post-hoc Steel–Dwass test; * *p* < 0.05: *** *p* < 0.001: n.s. not significant). Open bars, retinal explants in wild-type (+/+) mouse embryos; filled bars, retinal explants in *Ngr1*-deficient (−/−) mouse embryos.

The elongating effect of LOTUS on neurites might be attributed to its antagonism for NgR1 because LOTUS acts as an NgR1 antagonist through the UA/EC domain of LOTUS [33]. To investigate whether the antagonism of LOTUS for NgR1 contributes to the neurite-elongating action of LOTUS, we conducted a neurite outgrowth assay using retinal explant neurons that were derived from wild-type and *Ngr1*-deficient mouse E13 embryos and cultured using His-AP-LOTUS as a culture substrate. The similarly elongated neurites on the His-AP-LOTUS substrate were observed in *Ngr1*-deficient retinal explant neurons, compared with wild-type retinal explant neurons (Figure 2A). No significant difference in the neurite length was found between wild-type and *Ngr1*-deficient retinal explant neurons cultured on His-AP-LOTUS substrate (Figure 2B), suggesting that NgR1 does not mediate LOTUS-promoted neurite outgrowth.

### 3.3. LOTUS Interacts with Ten-4

**Figure 3:**
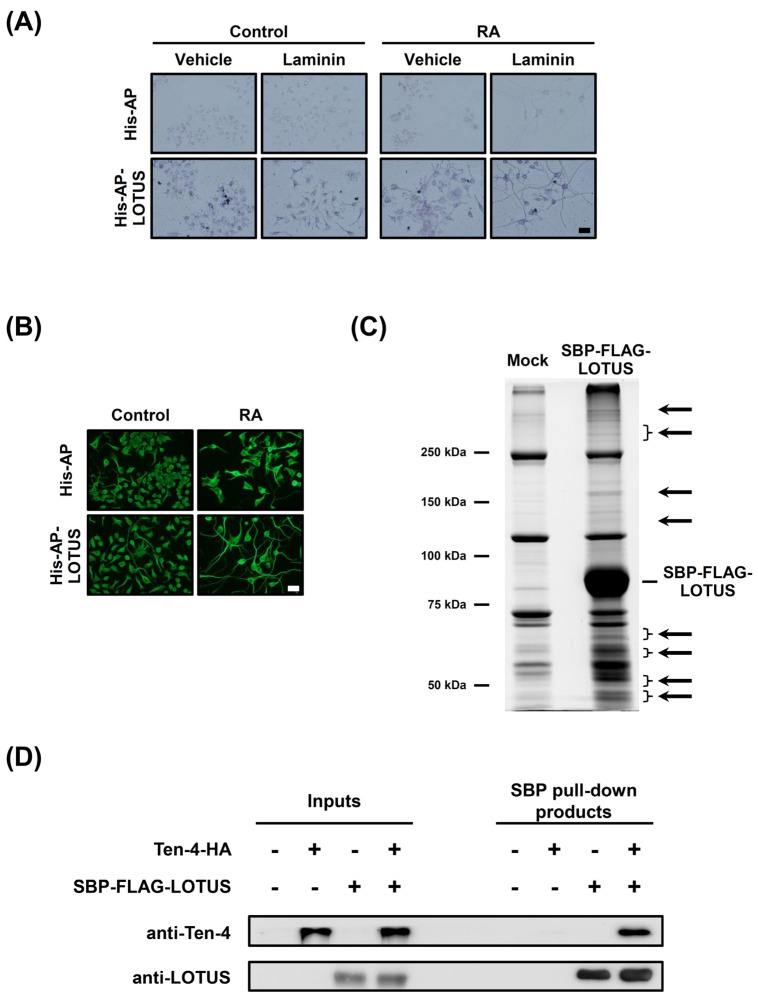
Identification of Ten-4 as a Binding Protein of LOTUS. (**A**) Detection of binding of His-AP or His-AP-LOTUS (25 nM) applied to Neuro2A cells cultured using vehicle or laminin as a culture substrate with or without RA treatment. Scale bars, 50 μm. (**B**) Immunostaining of α-tubulin with anti-α-tubulin antibodies in Neuro2A cells cultured using His-AP or His-AP-LOTUS (25 nM) as a culture substrate with or without RA treatment. Scale bars, 50 μm. (**C**) SYPRO Ruby staining of lysates from SBP-FLAG-LOTUS-overexpressed Neuro2A cells separated by SDS-PAGE. The gel pieces including the bands indicated by the arrows were excised and analyzed using LC-MS/MS. (**D**) Western blotting analysis using anti-Ten-4 or anti-LOTUS antibodies in the lysates of Ten-4-HA- and/or SBP-FLAG-LOTUS-overexpressed COS7 cells and in the pull-down products obtained from these cell lysates using streptavidin resins.

To search for new LOTUS-binding protein(s) that mediates the elongating effect of LOTUS on neurites, we attempted to find cell lines that express LOTUS-binding protein(s) and elongate their neurites when cultured using LOTUS as a culture substrate. Neuro2A cells were bound by His-AP-LOTUS with or without RA treatment that induces differentiation (Figure 3A) and elongated their neurites on a His-AP-LOTUS substrate with RA treatment (Figure 3B), suggesting that Neuro2A cells could express protein(s) which mediates neurite outgrowth induced by LOTUS-binding. To identify the binding protein(s) of LOTUS, we purified proteins interacting with LOTUS from the lysates of SBP-FLAG-LOTUS-overexpressed Neuro2A cells, followed by SDS-PAGE and fluorescent staining. More or higher-intensity bands were observed in the SBP-FLAG-LOTUS pull-down product (Figure 3C). We further conducted in-gel digestion and LC-MS/MS on the proteins including these bands. LC-MS/MS analysis determined enormous candidate LOTUS-binding molecules. From these candidates, we selected membrane proteins having the following characteristics as potential candidates (Table 1). The membrane proteins possess at least one extracellular domain and belong to extracellular matrix molecules, neurotrophic factors, or cell adhesion molecules, which regulate neurite outgrowth [1,2,3,4,5,6] because the LOTUS-binding molecule(s) is expected to mediate neurite outgrowth promoted by LOTUS extracellularly treated to cultured retinal explant neurons (Figure 1).

We first focused on Protocadherin-16, which is also categorized as a cell adhesion molecule and regulates neuronal migration [34,35], and Ten-3 shown to have higher score in the potential candidates (Table 1). To examine whether Protocadherin-16 or Ten-3 affects the elongating activity of LOTUS on neurites in RA-treated Neuro2A cells, we conducted a neurite outgrowth assay in Neuro2A cells that were treated with RA, cultured using His-AP-LOTUS as a culture substrate, and transfected with DsiRNA of *Protocadherin-16* or *Ten-3*. The neurite length was unaffected by DsiRNA transfection of *Protocadherin-16* or *Ten-3* in RA-treated Neuro2A cells without a His-AP-LOTUS substrate (Figure S1). His-AP-LOTUS elongated neurites in RA-treated Neuro2A cells transfected with a negative control DsiRNA and no significant differences in neurite length were observed between a negative control and *Protocadherin-16* or *Ten-3* transfected Neuro2A cells that were treated with RA and cultured on His-AP-LOTUS (Figure S1), suggesting that Protocadherin-16 or Ten-3 does not mediate the elongating activity of LOTUS on neurites.

We next focused on Ten-4, which is another protein having a higher score in the potential candidates (Table 1). To confirm the interaction of LOTUS with Ten-4 in LC-MS/MS analysis (Figure 3C and Table 1), we conducted a pull-down assay using Ten-4-HA- and/or SBP-FLAG-LOTUS-overexpressed COS7 cells and western blotting using each antibody. Overexpression of Ten-4-HA and/or SBP-FLAG-LOTUS in the cell lysates was confirmed (Figure 3D). No bands were observed in the pull-down products using the cells that did not overexpress Ten-4-HA or SBP-FLAG-LOTUS or that overexpressed Ten-4-HA only (Figure 3D). Streptavidin resins precipitated SBP-FLAG-LOTUS in the cells overexpressing SBP-FLAG-LOTUS together with or without Ten-4-HA (Figure 3D). The precipitates of Ten-4-HA were detected in the cells overexpressing Ten-4-HA and SBP-FLAG-LOTUS (Figure 3D). Thereafter, LOTUS interacted with Ten-4.

### 3.4. Knockdown of Ten-4 Suppresses LOTUS-Elongated Neurites in RA-Treated Neuro2A Cells

**Figure 4:**
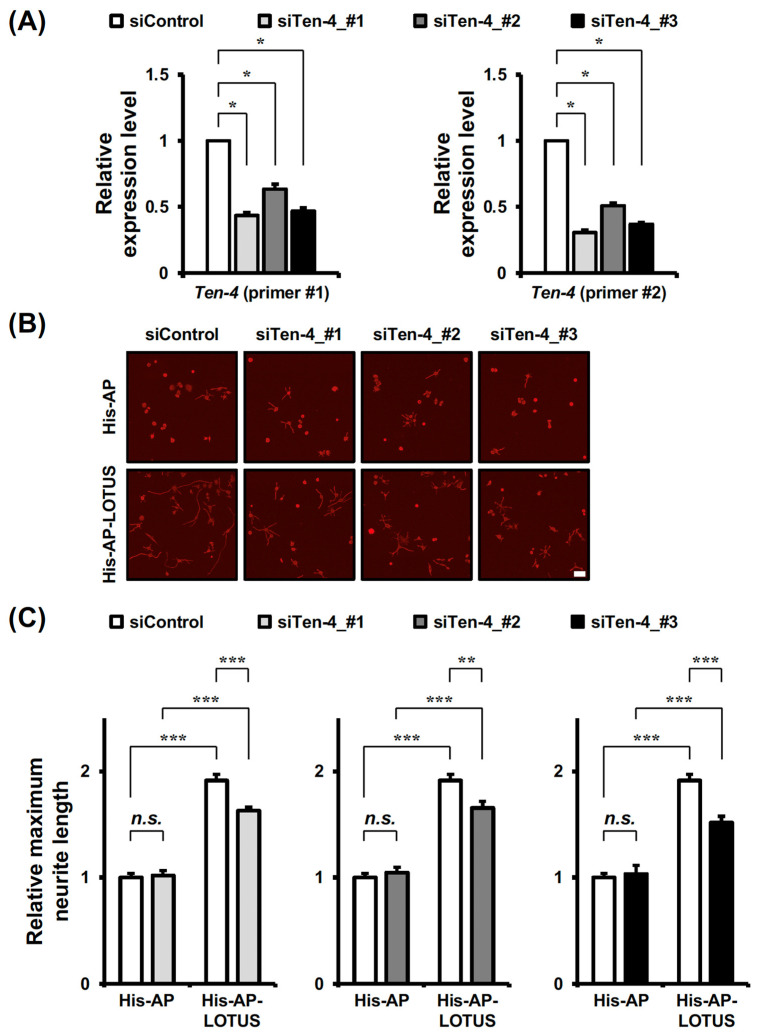
Inhibitory action of *Ten-4* knockdown on LOTUS-elongated neurites in RA-treated Neuro2A cells. (**A**) Quantification of *Ten-4* mRNA expression and the expression changes by DsiRNA in RA-treated Neuro2A cells. The expression levels of *Ten-4* in RA-treated Neuro2A cells transfected with each *Ten-4* DsiRNA were analyzed by RT-quantitative PCR using each primer set (*Beta-actin*, *Ten-4*_#1 and *Ten-4*_#2), calculated with those in the cells transfected with a negative control DsiRNA, normalized to the expression level of *Beta-actin* in the cells transfected with each *Ten-4* DsiRNA calculated with that in the cells transfected with a negative control DsiRNA, and presented as mean ± SEM from five independent cultures (*n* = 5 batches, Kruskal-Wallis test followed by post-hoc Steel test; * *p* < 0.05). White bars, cells transfected with a negative control DsiRNA; light gray bars, cells transfected with DsiRNA of *Ten-4* (#1); dark gray bars, cells transfected with DsiRNA of *Ten-4* (#2); black bars, cells transfected with DsiRNA of *Ten-4* (#3). (**B**) Immunostaining of β-tubulin with anti-β-tubulin antibodies in Neuro2A cells treated with RA, cultured using His-AP or His-AP-LOTUS (25 nM) as a culture substrate, and transfected with DsiRNA of a negative control or each *Ten-4*. Scale bar, 100 μm. (**C**) Quantification of neurite length in (**B**). The distance from an initial neurite segment to the neurite tip in each cell was measured as the neurite length in the Neuro2A cell. The maximum neurite length was shown as the single or longest neurite in each cell, normalized to the neurite length in the cell cultured on a His-AP substrate and transfected with a negative control DsiRNA, and presented as mean ± SEM from eight independent cultures (*n* = 8 batches, one-way factorial ANOVA test followed by post-hoc Tukey-Kramer test; ** *p* < 0.01: *** *p* < 0.001: n.s. not significant). White bars, cells transfected with a negative control DsiRNA; light gray bars, cells transfected with DsiRNA of *Ten-4* (#1); dark gray bars, cells transfected with DsiRNA of *Ten-4* (#2); black bars, cells transfected with DsiRNA of *Ten-4* (#3).

To address whether Ten-4 is associated with the elongating activity of LOTUS on neurites, we first analyzed the expression of Ten-4 in RA-treated Neuro2A cells with or without *Ten-4* DsiRNAs transfection. *Ten-4* mRNA was detected in RA-treated Neuro2A cells transfected with a negative control DsiRNA (Figure 4A), and the expression levels were reduced in RA-treated Neuro2A cells transfected with each DsiRNA of *Ten-4* (Figure 4A). Therefore, *Ten-4* was expressed in RA-treated Neuro2A cells, and *Ten-4* DsiRNA showed a silencing effect on *Ten-4* expression in the cells.

Next, we examined whether Ten-4 mediates the promoting effect of LOTUS on neurites in RA-treated Neuro2A cells. To address this issue, we conducted a neurite outgrowth assay using Neuro2A cells that were treated with RA, cultured using His-AP-LOTUS as a culture substrate, and transfected with each *Ten-4* DsiRNA. The neurite length in RA-treated Neuro2A cells was unchanged by *Ten-4* DsiRNA transfection without a His-AP-LOTUS substrate (Figure 4B,C). His-AP-LOTUS promoted neurites in RA-treated Neuro2A cells transfected with a negative control DsiRNA (Figure 4B,C), but His-AP-LOTUS-promoted neurites were inhibited in RA-treated Neuro2A cells transfected with each *Ten-4* DsiRNA (Figure 4B,C). Hence, Ten-4 mediated LOTUS-elongated neurites in RA-treated Neuro2A cells.

We also examined the promoting effect of LOTUS on neurites in RA-treated Neuro2A cells transfected with DsiRNA of *Protocadherin-16* or *Ten-3*. Knock down of *Protocadherin-16* or *Ten-3* did not cancel the LOTUS-induced neurite outgrowth (Figure S1). These results highlight the role of Ten-4 as a key molecule involved in neurite elongation induced by LOTUS.

### 3.5. Masking of LOTUS Substrate with a Soluble Form of Ten-4 Attenuates the Elongating Activity of LOTUS on Neurite Outgrowth in Retinal Explant Neurons

**Figure 5:**
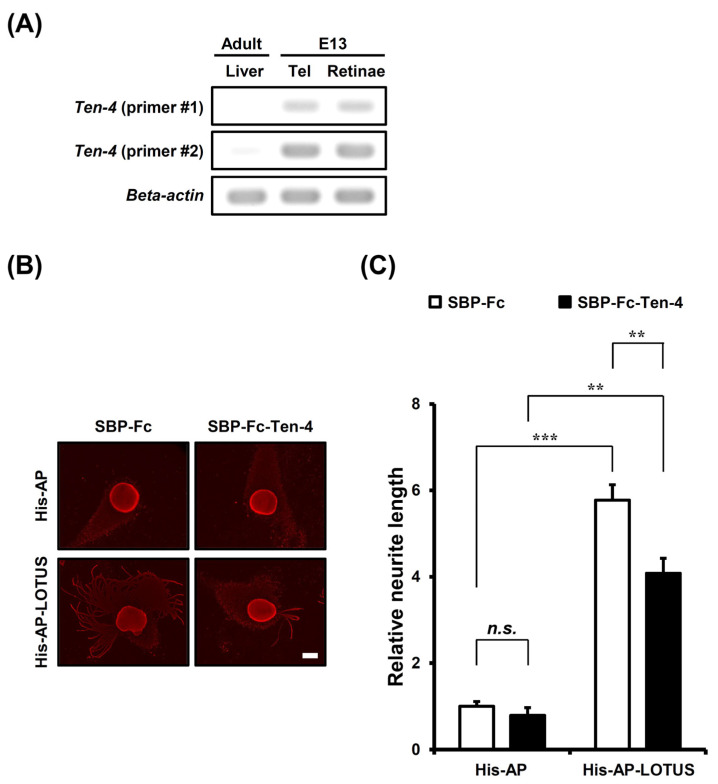
Suppressive activity of soluble form Ten-4 on LOTUS-promoted neurite outgrowth in retinal explant neurons. (**A**) RT-PCR analysis of *Ten-4* mRNA expression in adult wild-type mouse liver and wild-type mouse E13 embryonic telencephalon and retinae. *Beta-actin* was used as an internal control. Tel, telencephalon. (**B**) Immunostaining of β-tubulin with anti-β-tubulin antibodies in wild-type mouse E13 embryonic retinal explant neurons cultured using His-AP (25 nM) or His-AP-LOTUS (25 nM) as a culture substrate pre-incubated with SBP-Fc (1500 nM) or SBP-Fc-Ten-4 (1500 nM). Scale bars, 200 μm. (**C**) Quantification of neurite length in (**B**). The neurite length of the retinal explant was shown as the liner distance from the center of an explant to the neurite tip in the outer neurites, minus the radius of the explant, normalized to the neurite length of the explant cultured on a His-AP substrate pre-incubated with SBP-Fc, and presented as mean ± SEM from four independent cultures (*n* = 6–42 explants, Kruskal-Wallis test followed by post-hoc Steel-Dwass test; ** *p* < 0.01: *** *p* < 0.001: n.s. not significant). Open bars, retinal explants cultured on a substrate with SBP-Fc pre-incubation; filled bars, retinal explants cultured on a substrate with SBP-Fc-Ten-4 pre-incubation.

To address the involvement of Ten-4 in the elongating activity of LOTUS on neurite outgrowth in retinal explant neurons, we first analyzed the expression of *Ten-4* in mouse embryonic retinae at E13. *Ten-4* transcript was detected in mouse E13 embryonic retinae (Figure 5A), showing that Ten-4 was expressed in mouse E13 embryonic retinae. To examine whether Ten-4 mediates the promoting effect of LOTUS on neurite outgrowth in retinal explant neurons, we next conducted a neurite outgrowth assay using mouse E13 embryonic retinal explant neurons cultured using His-AP-LOTUS as a culture substrate that was pre-incubated with SBP-Fc-Ten-4. At this embryonic time, glial cells have no existence in the retinae [32]. The neurite length was not influenced in retinal explant neurons cultured on a His-AP substrate with SBP-Fc-Ten-4 pre-incubation (Figure 5B,C). Retinal explant neurons elongated their neurites on a His-AP-LOTUS substrates in the absence of SBP-Fc-Ten-4 pre-incubation (Figure 5B,C). In contrast, His-AP-LOTUS-promoted neurite outgrowth was attenuated in retinal explant neurons cultured on the substrate with SBP-Fc-Ten-4 pre-incubation (Figure 5B,C). The data indicate that masking of LOTUS by pre-incubation of soluble-form Ten-4 attenuates neurite outgrowth. Therefore, Ten-4 mediated LOTUS-promoted neurite outgrowth in retinal explant neurons.

## 4. Discussion

Neurite outgrowth underlies the wiring of the nervous system during development and regeneration following injury. LOTUS contributes to neuronal circuit formation in the developing lateral olfactory tract [18] and promotes axonal regeneration in the injured CNS [16,17,31,36,37]. These functions are attributed to the blockade of NgR1 and/or PIR-B by LOTUS [18,26,27,28]. In search for other functions of LOTUS, we identified Ten-4 as a new binding molecule of LOTUS and found the promoting effect of LOTUS on neurite outgrowth through its interaction with Ten-4.

Ten-4 is expressed in oligodendrocytes at the postnatal stage, interacts with each of the four Teneurin family members through their extracellular domain, and plays important roles in oligodendrocyte differentiation and myelination of axons in the CNS [8,13]. Ten-1, Ten-2, and Ten-3 are expressed in the postnatal CNS [10,11]. These reports suggest that the cellular functions of Ten-4 are exerted by the homophilic binding to Ten-4 expressed in the oligodendrocytes or by the heterophilic binding to the other three Teneurin family members that might be expressed in the oligodendrocytes, although it is unknown whether the three Teneurin family members except for Ten-4 are also expressed in the oligodendrocytes. On the other hand, Ten-4 is also expressed in the CNS neurons at the postnatal stage [13], leading to the possibility that its interaction with Ten-4 causes the cellular functions of Ten-4 expressed not only in the oligodendrocytes but also in the CNS neurons. In comparison with the roles in oligodendrocytes, it is undetermined whether Ten-4 expressed in the CNS neurons participates in neuronal functions through its interaction with Ten-4 expressed in the CNS neurons or the other neural cells, or with the three Teneurin family members expressed in the CNS, although Ten-4 expressed in Neuro2A cells mediates elongation of their neurites [15]. In the present study, we showed that Ten-4 interacts with LOTUS (Figure 3), which is expressed in the CNS neurons [18,38,39]. Therefore, there is a possibility that LOTUS may regulate oligodendrocyte differentiation by binding to Ten-4 expressed in the oligodendrocytes. Furthermore, we also found that Ten-4 is expressed in retinae and mediates LOTUS-induced neurite outgrowth through the binding of LOTUS (Figure 5). This finding is the first report to show that Ten-4 functions as a receptor that mediates promotion of neurite outgrowth in neurons. However, it remains to be elucidated whether the promoting effect by LOTUS-binding to Ten-4 expressed in the retinal explant neurons contributes to neuronal circuit formation in the optic nerve, optic chiasm, and optic tract in vivo. It will also be interesting to examine the involvement of the interaction between LOTUS and Ten-4 in oligodendrocyte differentiation or neuronal circuit formation in the developing and regenerating CNS.

Ten-4 mediates the promotion of neurite outgrowth by transducing the signal to FAK and thereby activating its downstream molecule Cdc42 [15], which is a member of Rho-family small G proteins [40]. In the present study, we showed that LOTUS promotes neurite outgrowth. This neurite outgrowth-promoting effect of LOTUS was unaffected in neurons derived from *Ngr1*-deficient mice (Figure 2) or treated with neutralizing antibodies against PIR-B, suggesting that LOTUS-promoted neurite outgrowth is independent of NgR1 or PIR-B, which is a LOTUS-binding molecule previously reported [18,28]. Furthermore, we found that Ten-4 mediates LOTUS-promoted neurite outgrowth. However, whether Ten-4 activates FAK or Cdc42 in the neurons with LOTUS-promoted neurite outgrowth is unknown. Further study is needed to uncover the molecular mechanisms of signal transduction by which LOTUS binding to Ten-4 promotes neurite outgrowth.

Previous studies have shown that missense mutations in the *TEN-4* gene are associated with Parkinson’s disease [41] and schizophrenia [42,43]. These mutations are mostly located in the extracellular domains of Ten-4 [41,42,43]. However, the molecular mechanisms by which the mutations lead to these disorders are unclear. The homophilic or heterophilic interaction of Ten-4 is exerted in their extracellular domains [8], and LOTUS is expected to interact with extracellular domains of Ten-4 because LOTUS is a transmembrane protein without any intracellular domains [18,33]. These mutations in the *TEN-4* gene might lose interactions between Ten-4 and the Teneurin family members or between Ten-4 and LOTUS, although the Teneurin family members- or the LOTUS-interacting site of Ten-4 is undetermined. Further investigation is required to verify whether the mutations in the *TEN-4* gene cause Ten-4 to change in binding affinity to the Teneurin family members or LOTUS.

Neurons establish functional networks in the developmental CNS by undergoing neuronal developmental processes such as migration, neurite outgrowth, and synapse formation [44] but fail to re-establish those in the adult mammalian CNS following injury. It is very important to recapitulate the developmental processes for effectively overcoming this failure [44]. Neurons in the adult spinal cord express Ten-4, and Ten-4 expression levels in the adult spinal cord are lower than those in the developmental spinal cord [13]. The decreased expression level of Ten-4 and/or the further reduction in expression level induced by injury might cause the regenerative failure of the axons after spinal cord injury, although whether Ten-4 is associated with reorganization of the functional neuronal circuits after spinal cord injury or whether Ten-4 expression level in the spinal cord is influenced by injury is not understood. We previously showed that LOTUS is a powerful antagonist for both receptors of NgR1 and PIR-B [18,26,27,28] that mediate axonal growth inhibition and thereby cause the failure of axonal regeneration after CNS injury [45,46]. In the present study, we identified Ten-4 as a new binding protein of LOTUS (Figure 3) and found that the interaction of LOTUS with Ten-4 promotes neurite outgrowth (Figure 4 and Figure 5). Our findings indicate that LOTUS not only rescues NgR1- and PIR-B-dependent inhibition of axonal growth but also promotes Ten-4-mediated neurite outgrowth, showing that LOTUS functions as a promising and potent factor for enhancing axonal regeneration following injury. In fact, we previously reported that LOTUS is down-regulated in injured spinal cord and brain infarct region [16,36] and demonstrated that overexpression or administration of LOTUS renders the CNS neurons to regenerate their axons in CNS-injured animal models such as optic nerve injury [16,31], spinal cord injury [16,17], ischemic stroke [36], and pyramidotomy [37]. This improved restorative capacity in the damaged CNS might be ascribed to the additive actions to suppress NgR1- and PIR-B-mediated inhibition of axonal growth and promote neurite outgrowth mediated by Ten-4. These findings may provide an additive mechanism by which LOTUS contributes to neuronal circuit formation during development and axonal regeneration in the adult CNS after injury.

## 5. Conclusions

We found a neurite-elongating action of LOTUS as a cellular function. This action was independent of NgR1, a LOTUS-binding protein previously identified [18], suggesting that this action is mediated by unidentified LOTUS-binding protein(s). A screening analysis using LC-MS/MS revealed that Ten-4 is a new LOTUS-binding partner. *Ten-4* knockdown and a soluble form of Ten-4 masking LOTUS substrate attenuated the neurite outgrowth-promoting effect of LOTUS. Taken together, LOTUS promotes neurite outgrowth through its binding to Ten-4. This new molecular mechanism of LOTUS to promote Ten-4-mediated neurite outgrowth may play an essential role in neuronal circuit formation in development and axonal regeneration after CNS injury.

## Figures and Tables

**Table 1:** The candidates of LOTUS-binding molecules.

Protein	Score	Coverage	# Unique Peptides	# Peptides	# Peptide Spectrum Matches	Molecular Weight [kDa]
Protocadherin-16	3.72	3.19	7	7	7	346.2
Teneurin-4	12.84	4.15	11	12	12	308.2
Teneurin-3	18.13	5.60	13	14	14	302.9
Integrin alpha-6	2.3	1.19	1	1	1	122.1
Integrin alpha-Iib	0	1.45	1	1	2	112.6

Potential candidates mediating neurite outgrowth promoted by LOTUS in LC-MS/MS analysis using LOTUS-pull-down products purified from Neuro2A cells overexpressing LOTUS.

## Data Availability

All data generated or analyzed during this study are included in this published article and its Supplementary Materials.

## Supplementary Materials

The following supporting information can be downloaded at: https://www.mdpi.com/article/10.3390/cells13161369/s1, Figure S1: Promoting effect of LOTUS on neurites in RA-treated Neuro2A cells transfected with DsiRNA of Protocadherin-16 or Ten-3.
