# Supplementary material for: Nogo Receptor Antagonist LOTUS Promotes Neurite Outgrowth through Its Interaction with Teneurin-4
Source: Cells. 2024 Aug 17;13(16):1369. doi: 10.3390/cells13161369 (PMC11352776; doi:10.3390/cells13161369)
Supplement: Supplementary file 1 [file cells-13-01369-s001.zip › cells-3119818-supplementary.pdf]

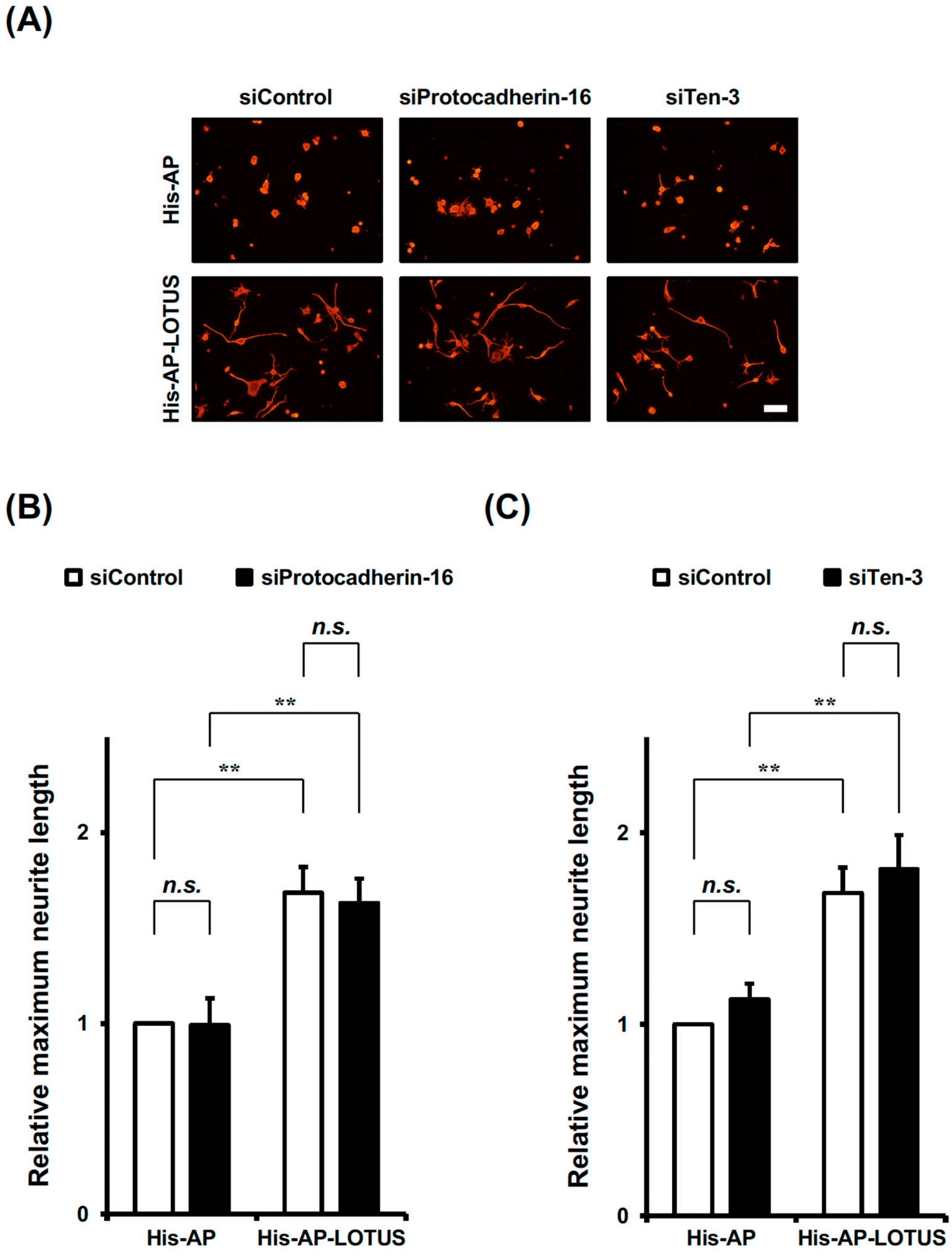

Figure S1. Promoting effect of LOTUS on neurites in RA-treated Neuro2A cells transfected with DsiRNA of Protocadherin-16 or Ten-3. (**A**) Immunostaining of βIII-tubulin with anti-βIII-tubulin antibodies in Neuro2A cells treated with RA, cultured using His-AP or His-AP-LOTUS (25 nM) as a culture substrate, and transfected with DsiRNA of a negative control, Protocadherin-16, or Ten-3. Scale bar, 100 μm. (**B**,**C**) Quantification of neurite length in RA-treated Neuro2A cells cultured on a substrate of His-AP or His-AP-LOTUS and transfected with Protocadherin-16 DsiRNA (**B**) or Ten-3 DsiRNA (**C**). The distance from an initial neurite segment to the neurite tip in each cell was measured as the neurite length in the Neuro2A cell. The maximum neurite length was shown as the single or longest neurite in each cell, normalized to the neurite length in the cell cultured on a His-AP substrate and transfected with a negative control DsiRNA and presented as mean ± SEM from four independent cultures (n = 4 batches, one-way factorial ANOVA test followed by post-hoc Tukey-Kramer test; ** $p < 0.01$, n.s., not significant). Open bars, cells transfected with a negative control DsiRNA; filled bars, cells transfected with Protocadherin-16 DsiRNA (**B**) or Ten-3 DsiRNA (**C**).
